# Miscellany

**Published:** 1892-07

**Authors:** 


### Miscellany.

---

### THE LAW GOVERNING THE PRACTICE OF MEDICINE IN NEW YORK STATE AND HOW IT IS ENFORCED.

The many inquiries directed to the Regents' office and to the secretaries of the various State Boards of Medical Examiners, indicate that the profession is largely interested in the operations of the law at present governing the practice of medicine in the State of New York. This manifested interest is accepted as of sufficient moment to warrant the publication of the salient features of the law, and, at the same time, to give the profession an insight into the methods and machinery necessary to its proper enforcement. The law, signed by the Governor June 4, 1890, went into effect September 1, 1891, and has been operative since that time. It provides: "From and after the 1st day of September, 1891, any person not theretofore lawfully authorized to practise medicine and surgery in this State, and desiring to enter upon such practice," may, after the following conditions have been fulfilled, receive an order to be examined before one of the three State Boards of Medical Examiners as to his medical qualifications. 1. Applicant must be more than twenty-one years of age. 2. Must present certificate of moral character from two legalized resident (State) medical practitioners. 3. Must be a graduated doctor of medicine from some legally incorporated medical college in the United States, or have received a diploma or license conferring the full right to practise all the branches of medicine or surgery in some foreign country. 4. Must have attended at least three full courses of lectures, in different years, in some legally incorporated *medical* college or colleges. 5. Must pay $25 into the treasury of the University of the State of New York. 6. Must present evidence of preliminary education as follows: Either *a*, usual academic degree; *b*, one year at academic-degree-conferring college; *c*, three years in a high school or academy: *d*, be in possession of Regents' medical student certificate; *e*, matriculation certificate required by present medical act of Canada; *f*, matriculation certificate from any university in Great Britain or Ireland; *g*, certificate of having passed examinations of any registered institution equivalent to one year in academic college or three years in high school.

All these preliminaries having been complied with, upon proof presented by the applicant, in his or her own handwriting, to the satisfaction of the Regents (on blank forms furnished on application), an order is given admitting the candidate to the next examination. Five regular examinations are held during the year (during the year 1892 there are still three regular examinations to be held as follows: June 14-17, September 27-30, and November 22-25,) simultaneously, at New York City (21 Cooper Union), Albany High School Building, Syracuse High School Building, and Buffalo High School Building, and as many special examinations are ordered as are deemed necessary by the Regents depending upon the exigencies which may arise. At these examinations the candidates are examined on the subjects of—1, Anatomy; 2, Physiology and Hygiene; 3, Chemistry; 4, Surgery; 5, Obstetrics; 6, Pathology and Diagnosis; and 7, Therapeutics, including Practice and Materia Medica. The candidate is allowed three hours' time in which to answer ten from among the fifteen questions submitted on each topic; each answer, if correct, has a value of ten points, and *each* full paper of ten questions answered must have a total value in markings of at least seventy-five points, otherwise the candidate is rejected. There are two sessions of three hours daily; each session devoted to one of the seven topics; thus, three and one-half days are requisite for completing the examination. The candidates are examined according to number, no name being allowed to appear on the answer papers; the name of the candidate is placed in an envelope marked with the corresponding number, is sealed, and left unopened until the final report of the examiners has been made.

There are three boards of State Medical Examiners, as follows: representing the Medical Society of the State of New York, Homeopathic Medical Society of the State of New York, and the Eclectic Medical Society of the State of New York.

*Medical Society of the State of New York.*—Physiology and Hygiene, William C. Wey, M. D., *President;* Chemistry and Materia Medica, Maurice J. Lewi, M. D., *Secretary*, 71 Lancaster street, Albany; Anatomy, William S. Ely, M. D.; Surgery, George Ryerson Fowler, M. D.; Obstetrics, William Warren Potter, M. D.; Pathology and Diagnosis, J. P. Creveling, M. D.; Theory and Practice and Therapeutics, Eugene Beach, M. D.

*Homeopathic Medical Society of the State of New York.*—Pathology and Diagnosis, Asa S. Couch, M. D., *President;* Anatomy, Horace M. Paine, M. D., *Secretary*, 105 State street, Albany; Physiology and Hygiene, A. R. Wright, M. D.; Chemistry, John McE. Wetmore, M. D.; Surgery, E. E. Snyder, M. D.; Obstetrics, William S. Searle, M. D.; Therapeutics, Practice and Materia Medica, Jay W. Sheldon, M. D.

*Eclectic Medical Society of the State of New York.*—Obstetrics, Hugh J. Linn, M. D., *President;* Surgery, Edwin S. Moore, M. D., *Secretary*, Bay Shore; Anatomy, William L. Tuttle, M. D.; Physiology and Hygiene, Robert Hamilton, M. D.; Chemistry, Harry B. Smith, M. D.; Pathology and Diagnosis, John P. Nolan, M. D.; Therapeutics, Practice and Materia Medica, *Vacancy.*

They are appointed by the Regents from nominations submitted by the State, Homeopathic, and Eclectic Medical Societies, for a term of three years, for every vacancy two names being submitted by the societies. The principal work of the examiners is to formulate questions for examination purposes and to mark the answers thereto. The questions submitted at each examination are the same for all candidates, excepting on the seventh topic (Therapeutics, Practice and Materia Medica), three sets of questions being furnished at each examination, each set representing the views of one of the three legally incorporated schools of medicine in the State on this subject, the candidate receiving the set for which he had expressed a wish in his original application for license. The questions to be used at the various examinations are decided upon as follows, those previously secured in a similar way having become exhausted: The Regents issue a notice requesting each of the twenty-one examiners to forward, on or before a certain date, sixty questions on the special topic to which each is assigned; subsequent to this date the Questions Board, consisting of six members, two from each board, is called in session. The questions on the seventh topic are handed to the two members representing their special board, who, as previously stated, and as particularly specified in the law, have complete charge of this subject. With the other six subjects, 180 questions having been submitted in each, the Questions Board acts as follows, taking Anatomy, for instance: The secretary reads a question alternately from each of the three papers submitted by the examiners in this topic, and unless each receives the unanimous vote of all present, it is stricken from the list of available questions. The 180 questions having passed through this process, are arranged in sets of fifteen, each set is numbered and sealed, and thus at one sitting, lasting, however, many hours, an average of ten complete sets of questions is prepared, thus providing for ten future examinations. These sets of questions are placed in the custody of the Regents, who, as the time for the next examination approaches, call upon the secretary of the Questions Board to review the printer's work after the questions are put up in type. The examinations proper are conducted by a sworn official of the Regents' office, who is not a member of any one of the State boards of medical examiners. As soon as the examinations are concluded, the answer papers are delivered to the secretary of the board selected by the candidate in his application, and by him in turn sent to the different individual examiners, who return the papers with their markings to the Board of Regents ; these answer papers thereupon become a part of the public records of the State. If a favorable report is made by all of the examiners on the answers of any applicant, a license is immediately forwarded to his address, thus enabling him to register at once and commence the practice of his profession. The last examination was concluded May 6th, and on May 14th the licenses were forwarded from the Regents' office to the successful candidates. Arrangements are now being made for the next examination, which will enable the Regents to forward these licenses within five days of the close of the examinations. The income accruing from this law goes to the Board of Regents, who, after paying all proper expenses, will, if ever there should be a surplus, apportion the money among the twenty-one examiners according to the number of candidates examined by each. Graduates in medicine who have been licensed by State examining boards of other States *of the United States only*, on convincing the Board of Regents that the standard of requirements adopted by the board of examiners which granted them the license is substantially the same as in New York State, may, upon the payment of $10, have such license endorsed. The following summary of the laws has been made :

1. The University of the State of New York is the only organization having authority to issue licenses to practise medicine in this State after September 1, 1891. These licenses must be registered by county clerks on application. (*Laws of New York*, 1890, ch. 507, § 8–9.)

2. Licenses issued before September 1, 1891, can be registered only as follows : *a*, A diploma granting the degree M. D., issued before September 1, 1891, by an incorporated medical college *in this State*, is a license to practise medicine and must be registered on application. (*Laws of New York*, 1889, ch. 647, § 2.)

*b*. A diploma granting the degree M. D., from a medical college *out of the State*, or a license to practise medicine in some foreign country, can be registered only if it was endorsed between June 18, 1880, and June 24, 1890, by an incorporated medical college of the State of New York, or by the University of the State of

New York, or if between June 24, 1890, and September 1, 1891, it was endorsed by the University of the State of New York. (*Laws of New York*, 1880, ch. 513, § 4; 1887, ch. 647, § 2; 1890, ch. 500.)

3. Students who had matriculated in a New York State medical college prior to June 5, 1890, and had not received the degree M. D. prior to September 1, 1891, to be exempt, must have filed a certificate with the University of the State of New York before August 4, 1891. (*Laws of New York*, 1891, ch. 311.)

Licenses of such candidates may be registered as follows: *a*, A diploma granting the degree M. D. from a New York State medical college, issued after September 1, 1891, can be registered on presentation of a certificate from the secretary of the University of the State of New York that the applicant had matriculated in some medical college of the State prior to June 5, 1890. (*Laws of New York*, 1891, ch. 311.)

*b*. A diploma granting the degree M. D. from a medical college not in the State, or license to practise in a foreign country, if endorsed by the University of the State of New York, can be registered on presentation of a certified copy of a certificate filed with the secretary of the University of the State of New York that the applicant had matriculated in some medical college of the State prior to June 5, 1890. (*Laws of New York*, 1891, ch. 311.)

All diplomas issued by medical colleges in this State prior to January 1, 1880, which are presented for registration after this date, should be referred to the University of the State of New York for examination before being registered.

And further, to quote the exact wording of the law:

§ 10. Nothing in this act shall be construed to interfere with or punish commissioned medical officers serving in the army or navy of the United States, or in the United States marine hospital service while so commissioned, or anyone while actually serving as a member of the resident medical staff of any legally incorporated hospital, or any legally qualified and registered dentist exclusively engaged in practising the art of dentistry, or interfere with manufacturers of artificial eyes, limbs, or orthopedical instruments or trusses of any kind from fitting such instruments on persons in need thereof; or any lawfully qualified physicians and surgeons residing in other States or countries, meeting registered physicians and surgeons of this State in consultation, or any physician or surgeon residing on the border of a neighboring State and duly authorized under the laws thereof to practise medicine or surgery therein, whose practice extends into the limits of this State; provided that such practitioner shall not open an office or appoint a place to meet patients or receive calls within the limits of the State of New York; or physicians duly registered in one county of this State, called to attend isolated cases in another county, but not residing or habitually practising therein.

Appended will be found the examination results thus far obtained:

| | |
|---|---|
| Number of those who fulfilled all requirements and received license | 34 |
| Number of applications still unacted upon | 10 |
| Rejected for failure to reach seventy-five per cent. at final medical examination | 1 |
| Deficient in preliminary education | 6 |
| Had never attended three full courses of lectures | 4 |
| License withheld because of moral reasons | 1 |
| Total number of applicants for license to practise medicine to date | 56 |

*Addenda.*—All examinations are conducted in English, unless the applicant expresses a desire to be examined in Latin. In that event, the application, with the reasons therefor, is placed before a committee consisting of the presidents of the three boards, whose decision is accepted by the Board of Regents. The candidate must pay the expenses of translation. Whenever it is found necessary to obtain the opinion of the board of examiners, the University authorities are requested to confer with a sub-committee of the conference, consisting of the president and secretary of each board, who are the executive committee of the boards. The boards proper meet twice in each year.

A syllabus is in course of preparation, and will be issued shortly. A candidate having failed, whether in one or all seven branches, his application for license is rejected. On re-examination no fee is exacted, but the candidate must pass the examination on all seven topics, regardless of the number he passed at the previous examination. Appeal for a re-opening of any examinations may be made to the Regents of the University.

Indications point to a class of from fifteen to twenty applicants at the next regular examination, June 14, 1892.

By order of the State Board of Medical Examiners, representing the Medical Society of the State of New York.

WILLIAM C. WEY, *President.*

Maurice J. Lewi, *Secretary.*

---

Girls Who Have Push.—There is an interesting group of bright girls at the New England Conservatory of Music, in Boston, who represent the quality of push characteristic of the American girl. There are some thirty-five of these girls, and they are being musically and vocally educated by *The Ladies' Home Journal* of Philadelphia. Some time ago this magazine offered, as a stimulant to girls to get subscriptions for it, free educations at the Conservatory. The American girl is quick to see a chance, and one by one these thirty-five girls have come from all parts of the country to Boston. They receive the very best the Conservatory affords, the most desirable rooms in the building are theirs, and they have all their wants carefully looked after by a wealthy periodical. Perhaps in no other country on the face of the globe could such a thing be possible. These girls, too, the reporter was told, belong to nice families, but they preferred to earn their own musical education rather than depend on the family purse. Of course, the particular girls are unknown to the scholars at large, and to all intents and purposes they are paying their own way. And they certainly are. It is said that the magazine is also educating a number of other girls at Wellesley, Smith, and Vassar Colleges.—*The Boston Journal.*

---

The Hudor Company, of Buffalo, manufactures for the use of the profession a lithia water which contains 41.37 grains of lithium carbonate to a gallon. It is highly charged with carbonic acid, and is an agreeable drink, besides being a useful medicine for the lithemic state and allied conditions. In addition, this company manufactures high grade ginger ale, lemon champagne, and other mineral waters, which they bottle and furnish to physicians and for private use. They also manufacture pure distilled water, which can be obtained by physicians at a very small cost, which is something of an object where a great quantity is required. It is a great advantage to a city to have such a manufactory within its own borders, and physicians should not be slow to manifest their appreciation of the fact.

---

Never judge an actor by the length of his hair. The property man sometimes has longer locks than the tragedian.—*Buffalo Express.*

---

Put off the presentation of your bill for a year, and the patient will conclude that your services were worth but little, and that you knew it.—*Ex.*

---

She found an explanation.—"You see, Mrs. Oilriz," said the suave young man, "they called them 'Canaanites' because they came from Canaan."

"Oh, I understand," said the old lady, affably. "There's something that Mr. Oilriz knowed and I didn't."

"Indeed!"

"Yes. He had heard that you spent several years in Paris, and he spoke of you yesterday "as a Parisite."—*Judge.*

